# Identification of potential key genes for HER-2 positive breast cancer based on bioinformatics analysis

**DOI:** 10.1097/MD.0000000000018445

**Published:** 2020-01-03

**Authors:** Yuxiang Lin, Fangmeng Fu, Jinxing Lv, Mengchi Wang, Yan Li, Jie Zhang, Chuan Wang

**Affiliations:** a Department of Breast Surgery, Fujian Medical University Union Hospital, Fuzhou, Fujian Province, China; b Bioinformatics and Systems Biology Graduate Program, University of California, San Diego, La Jolla, CA.

**Keywords:** bioinformatics analysis, differentially expressed genes, HER-2 positive breast cancer, hub genes

## Abstract

**Backgrounds:**

HER-2 positive breast cancer is a subtype of breast cancer with poor clinical outcome. The aim of this study was to identify differentially expressed genes (DEGs) for HER-2 positive breast cancer and elucidate the potential interactions among them.

**Material and methods:**

Three gene expression profiles (GSE29431, GSE45827, and GSE65194) were derived from the Gene Expression Omnibus (GEO) database. GEO2R tool was applied to obtain DEGs between HER-2 positive breast cancer and normal breast tissues. Gene ontology (GO) annotation analysis and Kyoto Encyclopedia of Genes and Genome (KEGG) pathway enrichment analysis was performed by the Database for Annotation, Visualization and Integrated Discovery (David) online tool. Protein-protein interaction (PPI) network, hub gene identification and module analysis was conducted by Cytoscape software. Online Kaplan–Meier plotter survival analysis tool was also used to investigate the prognostic values of hub genes in HER-2 positive breast cancer patients.

**Results:**

A total of 54 upregulated DEGs and 269 downregulated DEGs were identified. Among them, 10 hub genes including CCNB1, RAC1, TOP2A, KIF20A, RRM2, ASPM, NUSAP1, BIRC5, BUB1B, and CEP55 demonstrated by connectivity degree in the PPI network were screened out. In Kaplan–Meier plotter survival analysis, the overexpression of RAC1 and RRM2 were shown to be associated with an unfavorable prognosis in HER-2 positive breast cancer patients.

**Conclusions:**

This present study identified a number of potential target genes and pathways which might impact the oncogenesis and progression of HER-2 positive breast cancer. These findings could provide new insights into the detection of novel diagnostic and therapeutic biomarkers for this disease.

## 1 Introduction

Breast cancer (BC) is one of the most commonly diagnosed malignancies and a major cause of cancer mortality in women worldwide.^[1]^ For the year 2019, it is estimated that in the United States approximately 268,660 female patients would be diagnosed with BC and 41,760 would die from it.^[2]^ HER-2 positive breast cancer is caused by the amplification of the ERBB2/NEU receptor tyrosine kinase and represent approximately 20% of breast carcinomas.^[3,4]^ HER-2 overexpression is related with an increased risk of disease recurrence and death in this breast cancer subtype,^[5]^ so patients with HER-2 positive breast cancer are treated with chemotherapy plus anti-HER2 inhibitors such as trastuzumab.^[6–9]^ Other innovative HER-2 targeting drugs including Lapatinib,^[10,11]^ Pertuzumab,^[12,13]^ and Trastuzumab-DM1 (TDM-1)^[14–16]^ have also been proved effective for HER-2 positive breast cancer and are available in clinical application now. Despite these advances in anti-HER2 target therapies, as well as optimized surgical procedures and chemo/radiotherapy, emergence of drug-resistant, relapse or metastasis still occur after adjuvant treatment. Therefore, there is an urgent necessity to discover the novel etiological factors and molecular mechanisms for the diagnostic and treatment strategies of HER-2 positive breast cancer.

The molecular pathogenesis of tumorigenesis could be contributed to epigenetic or transcriptional alterations and somatic mutations. Aberrant genetic mutations in gene expression might lead to the malignant transformation of breast cancer. With the continuous improvement of sequencing and high-throughput DNA microarray analyses, numerous differentially expressed genes (DEGs) have been proved to be associated with the oncogenesis and progression of tumors. Therefore, identifying DEGs and elucidating the interactions among them is essential for the detection of novel diagnostic and therapeutic biomarkers for HER-2 positive breast cancer.

## 2 Material and methods

### 2.1 Datasets

The gene expression profiles analyzed in this study were obtained from the GEO (The Gene Expression Omnibus) database (https://www.ncbi.nlm.nih.gov/geo/). A total of 2150 series about human breast cancer and expression profiling by array were retrieved from the database. After a careful review, three gene expression profiles (GSE29431, GSE45827, and GSE65194) were chosen, of which all expression profiles were based on GPL570 platform [HG-U133_Plus_2] Affymetrix Human Genome U133 Plus 2.0 Array. Among them, the GSE29431 dataset includes 28 HER-2 positive breast cancer samples and 12 normal tissues samples, while the GSE45827 and GSE65194 expression profiles were from the same specimens and consist of 30 HER-2 positive breast cancer samples and 11 matched normal breast tissues.

### 2.2 Data processing of DEGs

GEO2R (http://www.ncbi.nlm.nih.gov/geo/geo2r/) is an online tool to screen genes that are differentially expressed across different groups of samples. The raw microarray data files between HER-2 positive breast cancer and normal breast tissues were subsequently conducted by GEO2R. The adjusted *P* value and |logFC| were carried out for each dataset, with adjusted *P* < .01 and |logFC|≥2.0 were considered as DEGs. The intersecting part was calculated using the Venn diagram webtool (bioinformatics.psb.ugent.be/webtools/Venn).

### 2.3 Functional and pathway enrichment analyses of the DEGs

Gene ontology (GO) analysis is a commonly used approach to provide functional classification for genomic data, including biological process (BP), molecular function (MF), and cellular component (CC).^[17]^ Kyoto Encyclopedia of Genes and Genomes (KEGG) database^[18]^ is a knowledge base for systematic analysis, annotation or visualization of gene functions and biological pathways. GO annotation analysis and KEGG pathway enrichment analysis of DEGs in the present study was analyzed by the Database for Annotation, Visualization and Integrated Discovery (David, http://david.abcc.ncifcrf.gov/) online tool.^[19]^*P* < .05 and gene counts >10 was considered statistically significant.

### 2.4 PPI network construction, hub gene identification and module analysis

The Search Tool for the Retrieval of Interacting Genes (STRING) database (http://string.embl.de/)^[20]^ is designed to analyze the protein-protein interaction (PPI) information. DEGs were mapped to the STRING database to evaluate the interactive relationships, with a combined score >0.9 defined as significant. Subsequently, the PPI network was visualized by Cytoscape software (www.cytoscape.org/).^[21]^ CytoHubba, a plugin in cytoscape, was applied to calculate the degree of each protein node and the top 10 genes were identified as hub genes. Moreover, the other plugin for Cytoscape, MCODE (The Molecular Complex Detection)^[22]^ was selected to screen the modules of the PPI network. The criteria was as follows: degree cutoff = 2, node score cutoff = 0.2, k-core = 2 and maximum depth = 100.

### 2.5 Survival analysis of hub genes

To investigate the prognostic values of hub genes in HER-2 positive breast cancer patients, the Kaplan–Meier plotter mRNA breast cancer database (http://kmplot.com/analysis/)^[23]^ was performed. Probes of genes were calculated based on the “only JetSet best probe set”. For each gene, patients were divided into two groups according to the “Auto select best cutoff ”. *P* *<* .05 was considered statistically significant.

### 2.6 Ethics and dissemination

The study protocol was approved by the Ethics Committee of Fujian Medical University Union Hospital and all participants provided written informed consent.

## 3 Results

### 3.1 Identification of DEGs

**Figure 1:**
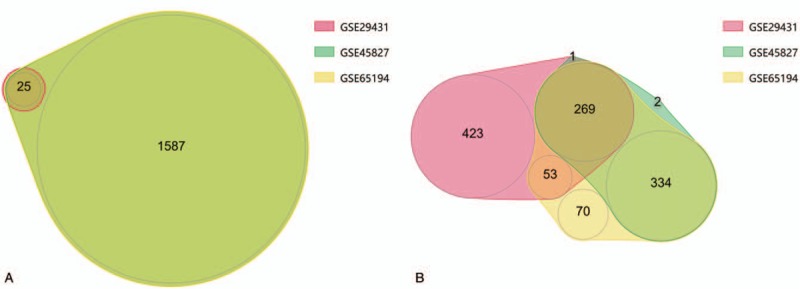
Venn diagram of DEGs obtained from 3 gene expression profiles. (A) Upregulated genes. (B) Downregulated genes.

Three gene expression profiles (GSE29431, GSE45827, and GSE65194) were selected in this study. Among them, GSE29431 includes 28 HER-2 positive breast cancer samples and 12 normal tissues samples, while GSE45827 and GSE65194 contain 30 HER-2 positive breast cancer samples and 11 matched normal breast tissues, respectively. Based on the GEO2R analysis and criteria of *P* < .01 and |logFC|≥2, 825 DEGs were obtained from GSE29431, including 79 upregulated genes and 746 downregulated genes. While for GSE45827 and GSE65194, 2218 DEGs (1612 upregulated genes, 606 downregulated genes) and 2338 DEGs (1612 upregulated genes, 726 downregulated genes) were identified. Venn diagram was subsequently applied to gain the intersection of the DEG profiles (Fig. 1), a total of 323 DEGs (54 upregulated genes and 269 downregulated genes) were indicated significant in HER-2 positive breast cancer tissues compared with normal tissues.

### 3.2 Functional and pathway enrichment analyses

All DEGs were uploaded to DAVID to identify significant GO categories and KEGG pathways. The results of GO analysis demonstrated that DEGs were markedly enriched in BP, including cell adhesion, angiogenesis and cell proliferation. Go CC analysis also showed that DEGs were enriched in proteinaceous extracellular matrix, focal adhesion, cell surface and basolateral plasma membrane. As for MF analysis, DEGs were significantly enriched in heparin binding, actin binding, protein kinase binding and calcium ion binding. In addition, the results of KEGG pathway analysis indicated that DEGs were mainly enriched in PPAR signaling pathway, pathways in cancer, focal adhesion and AMPK signaling pathway (Table 1).

**Table 1:**
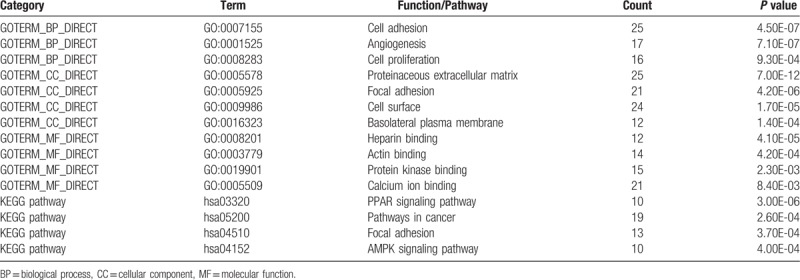
Gene ontology and KEGG pathway analysis of DEGs associated with HER-2 positive breast cancer.

### 3.3 PPI network construction, modules selection and hub gene identification

**Figure 2:**
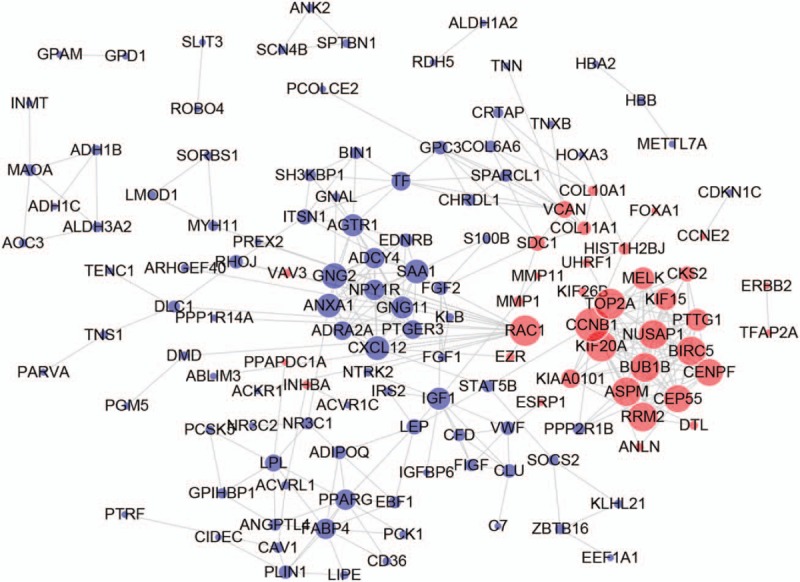
Protein-protein interaction network demonstrated with the DEGs.

**Figure 3:**
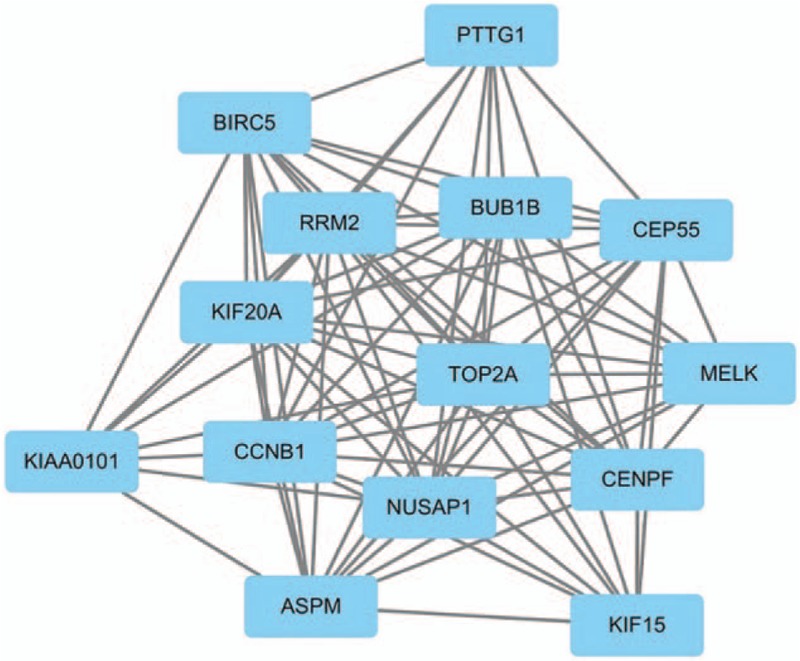
Module analysis constructed from the PPI network.

The PPI network of DEGs were constructed in the STRING database (version 10.5) and visualized by Cytoscape. With a combined score >0.9 defined as significant, a total of 299 nodes and 277 edges were evaluated in the PPI network (Fig. 2). The top ten genes demonstrated by connectivity degree in the PPI network were Cyclin B1 (CCNB1), Ras-related C3 botulinum toxin substrate 1 (RAC1), DNA topoisomerase 2-alpha (TOP2A), Kinesin family member 20A (KIF20A), Ribonucleotide reductase regulatory subunit M2 (RRM2), Abnormal spindle microtubule assembly (ASPM), Nucleolar and spindle associated protein 1 (NUSAP1), Baculoviral IAP repeat containing 5 (BIRC5), BUB1 mitotic checkpoint serine/threonine kinase B (BUB1B) and Centrosomal protein 55 (CEP55), relevant results were shown in Table 2 and all hub genes were upregulated in HER-2 positive breast cancer. A significant module including 14 nodes and 91 edges was also constructed from the PPI network by MCODE (Fig. 3).

**Table 2:**
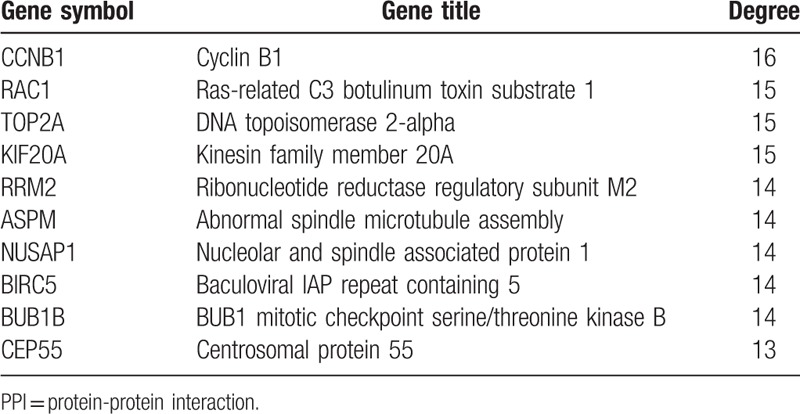
Top ten genes demonstrated by connectivity degree in the PPI network.

### 3.4 Survival analysis of the identified hub genes

**Figure 4:**
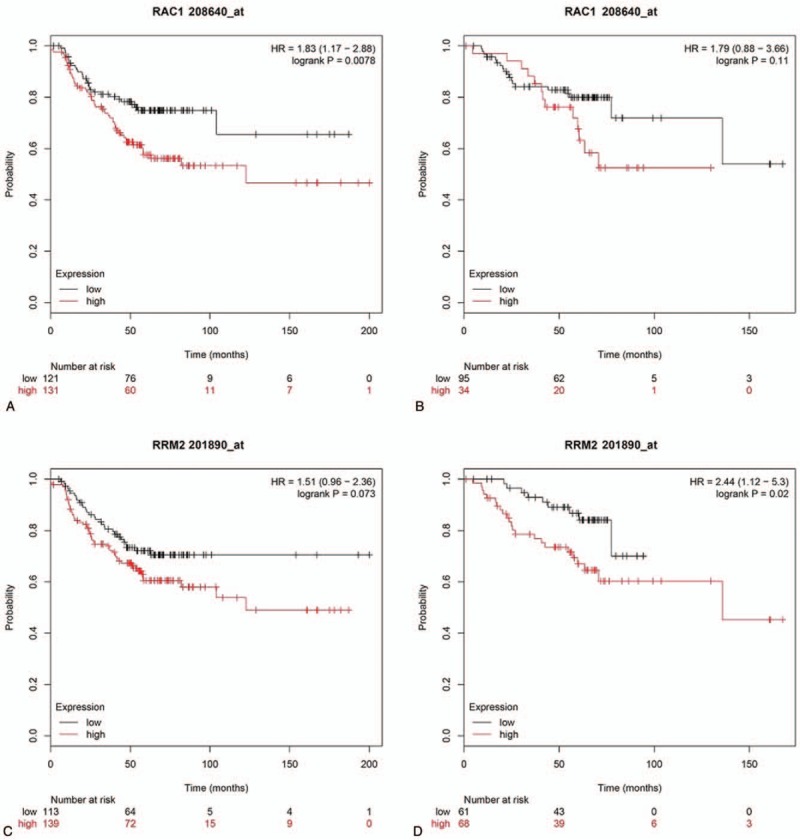
Kaplan–Meier survival analysis for the 10 potential hub genes in HER-2 positive breast cancer patients. (A) Relapse free survival for RAC1 expression. (B) Overall survival for RAC1 expression. (C) Relapse free survival for RRM2 expression. (D) Overall survival for RRM2 expression.

To evaluate the prognostic roles of the ten potential hub genes with HER-2 positive breast cancer, the Kaplan–Meier plotter bioinformatics analysis platform was applied. A total of 416 HER-2 positive breast cancer patients were available for the analysis of relapse free survival (RFS) and overall survival (OS). Higher expression of RRM2 was associated with a worse OS (HR = 2.44; 95% CI = 1.12–5.30, *P* = .02) but not RFS (HR = 1.51; 95% CI = 0.96–2.36, *P* = .073), while the overexpression of RAC1 was an unfavorable prognostic factor of RFS (HR = 1.83; 95% CI = 1.17–2.88, *P* = .0078) but not OS (HR = 1.79; 95% CI = 0.88–3.66, *P* = .11) (Fig. 4).

## 4 Discussion

Breast cancer is a heterogeneous disease in which the biological features and clinical behaviors vary from each subtype. HER-2 positive breast cancer is caused by the amplification of the ERBB2/NEU receptor and associated with an increased risk of disease recurrence and death. Despite advances in current therapeutics such as anti-HER2 therapy, relapse or metastasis still occur after adjuvant treatment. Further understanding in etiological and molecular mechanisms of HER-2 positive breast cancer could offer a great number of potential clues in developing novel therapeutic agents.

In this study, gene expression profilings were extracted from GEO databases to identify potential key genes related with HER-2 positive breast cancer. DEGs between HER-2 positive breast cancer and normal breast tissues were conducted by GEO2R, 54 upregulated genes and 269 downregulated genes were identified in total. These DEGs were shown to be mostly involved in cell adhesion, angiogenesis and cell proliferation for the GO BP term analysis and conformed our knowledge that these factors were of vital importance for tumor development and progression.^[24–28]^ Moreover, the DEGs were found significantly enriched in KEEG pathways of PPAR signaling pathway, pathways in cancer, focal adhesion and AMPK signaling pathway. PPAR signaling pathway was indicated to be a potential predictor of neoadjuvant chemotherapy response in breast cancer.^[29]^ While numerous studies have demonstrated that targeting focal adhesion kinase could improve trastuzumab response and might be an effective measure to overcome trastuzumab resistance in HER-2 positive breast cancer.^[30,31]^ In addition to these, AMPK was found dysfunctional in breast cancer, with the reduced signaling via the AMPK pathway was correlated with a higher histological grade and axillary node metastasis of breast cancer.^[32]^

PPI network and module analysis was also conducted to evaluate the associations of the DEGs, 10 hub genes were revealed, including CCNB1, RAC1, TOP2A, KIF20A, RRM2, ASPM, NUSAP1, BIRC5, BUB1B, and CEP55. Despite there were more downregulated DEGs identified, all of these genes were found to be upregulated in HER-2 positive breast cancer. In the Kaplan–Meier plotter bioinformatics analysis, higher expression of RAC1 and RRM2 were indicated to be an unfavorable prognostic factor for HER-2 positive breast cancer patients. However, the sample size of this survival analysis was still not large enough, which may lead to the limited statistical power and impact on the precision and accuracy of results, additional population-based studies are still necessary to validate the findings.

RAC1 is a member of the Rho GTPase family, which mainly regulates the assembly and disassembly of cytoskeletal elements.^[33]^ Rho GTPases were shown to be correlated with various tumorigenic process, such as angiogenesis, cell transformation, invasion and metastasis.^[34,35]^ RAC1 was proved to be dysregulated in both expression and activity in a variety of tumor cells.^[36]^ The downregulation of RAC1 was indicated to generate the inhibition of migration in colorectal adenocarcinoma,^[37]^ with an increased expression of RAC1 was associated with decreased cancer cell differentiation and advanced pathological stage for breast cancer.^[38]^ Also, RAC1 GTPase promotes the survival of breast cancer cells in response to hyper-fractionated radiation treatment. Besides, in HER2-positive breast cancers, high expression of RAC1 mRNA significantly correlated with poor prognosis of the patients. In our study, RAC1 was found to be upregulated in HER-2 positive breast cancer, while it was interacted more with the downregulated genes (shown in Fig.). Numerous evidences have suggested that RAC1 could emerge as a critical role in tumor for its angiogenic and invasive behaviors. The activity of RAC1 in endothelial cells was demonstrated essential for vascular development and could serve as promising therapeutic target for the treatment of human diseases involving aberrant neovascularization.^[39]^ The patterns of metastatic spread in cancer cells were mainly attributed to the stroma, endothelium and extracellular matrix, while RAC1 played a role in the formation of cell-cell adhesions and also took part in determining these patterns as well.^[40]^ Further studies on the biological function of RAC1 in the tumor microenvironment, both on the cancer cells or on the surrounding stromal and endothelial cells, could help us gain more insight into the alternative therapeutic targets for tumor angiogenesis and metastasis. RRM2 is a key gene in pyrimidine metabolism and has been proved to be highly up-regulated in breast cancer patients.^[41]^ Relevant studies also suggested RRM2 as a prominent marker for breast cancer metastasis^[42]^ and could play a crucial role in tamoxifen resistance.^[43]^ CCNB1 is well known for its critical role in regulating Cyclin-dependent kinase 1 (Cdk1), which initiates the process from G2 phase to mitosis.^[44]^ Overexpression of CCNB1 is indicated to be associated with aggressive phenotype and poor prognosis for breast cancer.^[45,46]^ Besides, the defective CCNB1 induction is also demonstrated to contribute to TDM1 acquired resistance in HER2-positive breast cancer.^[47]^ TOP2A is located in a separate amplicon downstream to HER2 and frequently expressed in HER2-positive breast cancer.^[48,49]^ The TOP2A aberration or CEP17 duplication was considered to be independently predictive of adjuvant anthracycline chemotherapy for early breast cancer.^[50]^ KIF20A is a member of KIFs superfamily which participate in cell mitosis and migration.^[51,52]^ It has been reported that KIF20A is overexpressed in breast cancer and could confer paclitaxel resistance.^[53–55]^ ASPM has been well studied and could play a potential molecular target in glioblastoma.^[56]^ However, little is known about the role of ASPM in breast cancer. A 4-gene predictive model including ASPM has been established and validated to predict response to endocrine therapy in breast cancer,^[57]^ future studies concerning ASPM and breast cancer are still necessary. NUSAP1 is an important mitotic regulator and the overexpression of NUSAP1 could result in the profound bundling of spindle microtubules.^[58]^ The aberrant expression of NUSAP1 has also been identified to be differed between ductal carcinoma in situ (DCIS) and invasive ductal carcinoma (IDC) and associated with a worse prognosis for breast cancer.^[59,60]^ BIRC5 (survivin) is a well-known member of the inhibitor of apoptosis family.^[61]^ The high expression of BIRC5 could directly inhibit the activity of caspase-3 and caspase-7, thus leading to the prevention of apoptosis.^[62,63]^ Numerous studies have confirmed the relationship between BIRC5 overexpression and survival in breast cancer patients.^[64–66]^ In addition, BIRC5 was also identified to be a prognostic factor for non-pCR breast cancer patients after neoadjuvant chemotherapy.^[67]^ BUB1B is a member of the SAC protein family and acts as a key component of the mitotic checkpoint.^[68]^ The overexpression of BUB1B was shown to be linked with chromosomal instability in breast cancer cells and cancer pathogenesis in gene expression profilings.^[69,70]^ CEP55 is also a key regulator of cytokinesis and its overexpression is connected with genomic instability.^[71]^ A high expression of CEP55 has been demonstrated as a determinant of cell fate during perturbed mitosis in breast cancer.^[72]^ However, the role of CEP55 in breast cancer is still not clear and required further research.

## 5 Conclusion

In this present study, we conducted a comprehensive bioinformatics analysis and revealed a number of potential target genes and pathways which might impact the oncogenesis and progression of HER-2 positive breast cancer. These findings had provided new insights into the diagnosis and treatment of this disease. However, the main limitation of this study is the lack of experimental validation. Therefore, additional population-based studies, together with larger sample sizes, as well as further functional studies, are still warranted to confirm our findings.

## Author contributions

**Data curation:** Fangmeng Fu.

**Formal analysis:** Yuxiang Lin.

**Funding acquisition:** Chuan Wang.

**Methodology:** Jinxing Lv.

**Software:** Mengchi Wang.

**Writing – original draft:** Yan Li.

**Writing – review & editing:** Jie Zhang.
